# Genomic insights into the genotype–environment mismatch and conservation units of a Qinghai–Tibet Plateau endemic cypress under climate change

**DOI:** 10.1111/eva.13377

**Published:** 2022-06-10

**Authors:** Heng Yang, Jialiang Li, Richard Ian Milne, Wenjing Tao, Yi Wang, Jibin Miao, Wentao Wang, Tsam Ju, Sonam Tso, Jian Luo, Kangshan Mao

**Affiliations:** ^1^ 12530 Key Laboratory of Bio‐Resource and Eco‐Environment of Ministry of Education College of Life Sciences State Key Laboratory of Hydraulics and Mountain River Engineering Sichuan University Chengdu China; ^2^ Institute of Molecular Plant Sciences The University of Edinburgh Edinburgh UK; ^3^ 91597 College of Science Tibet University Lhasa China; ^4^ Tibet Key Laboratory of Forest Ecology in Plateau Area of Ministry of Education Research Institute of Tibet Plateau Ecology National Key Station of Field Scientific Observation & Experiment of Alpine Forest Ecology System in Nyingchi Tibet Agriculture & Animal Husbandry University Nyingchi China

**Keywords:** climate change, conservation units, genomic vulnerability, King Cypress, local adaptation, Qinghai–Tibetan Plateau

## Abstract

Habitat loss induced by climate warming is a major threat to biodiversity, particularly to threatened species. Understanding the genetic diversity and distributional responses to climate change of threatened species is critical to facilitate their conservation and management. *Cupressus gigantea*, a rare conifer found in the eastern Qinghai–Tibet Plateau (QTP) at 3000–3600 m.a.s.l., is famous for its largest specimen, the King Cypress, which is >55 m tall. Here, we obtained transcriptome data from 96 samples of 10 populations covering its whole distribution and used these data to characterize genetic diversity, identify conservation units, and elucidate genomic vulnerability to future climate change. After filtering, we identified 145,336, 26,103, and 2833 single nucleotide polymorphisms in the whole, putatively neutral, and putatively adaptive datasets, respectively. Based on the whole and putatively neutral datasets, we found that populations from the Yalu Tsangpo River (YTR) and Nyang River (NR) catchments could be defined as separate management units (*MU*s), due to distinct genetic clusters and demographic histories. Results of gradient forest models suggest that all populations of *C*. *gigantea* may be at risk due to the high expected rate of climate change, and the NR *MU* had a higher risk than the YTR *MU*. This study deepens our understanding of the complex evolutionary history and population structure of threatened tree species in extreme environments, such as dry river valleys above 3000 m.a.s.l. in the QTP, and provides insights into their susceptibility to global climate change and potential for adaptive responses.

## 1 INTRODUCTION

Rapid climate change driven by human activities is one of the leading causes of global biodiversity loss in this new millennium (Thomas et al., 2004). Therefore, understanding how species will respond to future climate change and predicting their extinction risks are crucial if conservation biologists are to implement conservation efforts to protect threatened species (Brook et al., 2008; Cronk, 2016; Miraldo et al., 2016; Scheffers et al., 2016; Urban, 2015). To avoid climate‐driven extinction, species must (a) escape to suitable climatic conditions, (b) acclimate via phenotypic plasticity, or (c) adapt via natural selection based on standing genetic variation and/or new mutations. However, acclimation is limited in its effect, and rates of both mutation and migration seem unlikely to keep pace with ongoing climate change, especially for species with long lifespans, such as forest tree species (Bisbing et al., 2020; Dauphin et al., 2021). Therefore, standing adaptive variation in the gene pool is crucial for long‐lived species that face rapid climate change in the near future (Bragg et al., 2015; Du et al., 2020).

Populations living under heterogeneous conditions can adapt to local environments, and candidate genes that contribute to local adaptation are typically those that show strong environmental gradients (Coop et al., 2010) or high differentiation (e.g., *F*_ST_) between distinct habitats (Foll & Gaggiotti, 2008). Candidate gene identification was previously limited by the use of traditional molecular markers (e.g., allozymes or microsatellites), as these are mostly neutral and/or provide relatively few variable single nucleotide polymorphisms (SNPs) to detect selected signals. However, high‐throughput sequencing makes it possible to simultaneously identify thousands of SNPs associated with important traits, fitness, or environmental factors, providing new insights into population genetic mechanisms involved in local adaptation (Allendorf et al., 2010; Hohenlohe et al., 2020). Moreover, population genomic data can also address with high precision various important scientific questions concerning the conservation and management of threatened species, such as demographic history, population structure, genetic differentiation, and genetic diversity (Hohenlohe et al., 2020; Li et al., 2020, 2021).

Also advancing is modeling work that translates local adaptation‐related genomic data into spatial inferences about populations threatened by climate change (Capblancq et al., 2020; Fitzpatrick & Keller, 2015). Species distribution models (SDMs) are empirical modeling tools widely used to generate predictions about the potential distributions of populations and species under future climate change scenarios (Bakkenes et al., 2002; Guisan & Thuiller, 2005). However, these models are often criticized for their oversimplistic assumptions and for neglecting critical biological processes such as adaptation to rapid climate change via selection upon existing genetic and phenotypic variation (Alberto et al., 2013; Benito Garzón et al., 2019). To address this issue, recent studies have focused on the effects and distributions of potentially adaptive variation associated with climate, in order to predict and account for genomic mismatches between modern and future environments (Bay et al., 2018; Fitzpatrick & Keller, 2015; Jia et al., 2020; Zhao et al., 2020), especially for forest trees (Fitzpatrick & Keller, 2015; Jia et al., 2020; Zhao et al., 2020). Such emerging methods can be used to define the extent of nature reserves, identify populations that are most likely to become extinct, and help to mitigate the threat of climate change (Aitken & Bemmels, 2016; Bay et al., 2017; Fitzpatrick & Keller, 2015).

*Cupressus gigantea*W.C. Cheng & L.K. Fu was once considered to be a variety of *C*. *torulosa* D. Don [*Cupressus torulosa* D. Don var. *gigantea* (W.C. Cheng et L.K.); Farjon, 2005], is a rare conifer species with remarkable ecological, ornamental, and religious value, and is endemic to the eastern Qinghai–Tibet Plateau *sensu lato* where it is distributed in the Yalu Tsangpo River and Nyang River valleys. One famous individual, the King Cypress, stands >55 m tall and is >3000 years old; it forms a focal point in a very important sacred forest for Tibetans and attracts many tourists every year. The species is also of great ecological value because it is a keystone species in the Yalu Tsangpo River valley between 3000 and 3600 m.a.s.l. It is found in scattered stands or groves, with few large shrubs, surrounded by open dry scrubland that is often seasonally grazed. In addition, it occurs in sandy or stony fluvio‐glacial sediments or on slopes over limestone, usually on the east‐facing slopes of the valley, where it represents a unique adaptation to extreme environments (Zhou et al., 2019). However, human activity has disturbed and fragmented its range (Farjon, 2005; Zheng & Fu, 1978), and it is consequently listed as “Class I” on the *Chinese National Protection List of Wild Plants* (Liguo Fu & Chin, 1992) and “Vulnerable” on the *IUCN* Red List (Zhang et al., 2013). Previous studies have mainly focused on its biological characteristics, community structure, propagation techniques (Xin et al., 2019; Yang, 2015; Zhang, 2006), and phylogenetic relationships with congeners (Little, 2006; Mao et al., 2012; Terry et al., 2018). Its genetic diversity has been investigated, but only using traditional markers such as intersimple sequence repeat amplification (ISSR), random amplified polymorphic DNA (RAPD), simple sequence repeats (SSRs), and chloroplast DNA sequences (Fu et al., 2019; Lu et al., 2014; Xia et al., 2008; Xu et al., 2010). In addition, conifer genomes are usually of huge sizes with complex structures (>8 Gb; Nystedt et al., 2013; Scott et al., 2020), and whole‐genome resequencing is therefore not cost‐effective for population genomic analyses (Azaiez et al., 2018; De La Torre et al., 2014). Some strategies, such as reduced‐representation genome sequencing (Jia et al., 2020), exome sequencing (Neves et al., 2013), or RNA‐seq (Ru et al., 2018), have been used to reduce genome complexity and applied in conifer population genomics in recent decades. Among them, RNA‐seq has already been used to characterize gene family expansions of *C*. *gigantea* (Zhou et al., 2019) and to reveal adaptive introgression from this species to its congeners (Ma et al., 2019). Therefore, RNA sequencing and in‐depth analyses are shown to be powerful approaches when studying the evolution and conservation of *C*. *gigantea*.

In this study, we analyzed RNA sequences of 96 individuals from 10 populations of *C*. *gigantea* to address the following questions: (a) How did past climate history impact the demographic history of *C*. *gigantea* and shape its current genetic diversity and distribution pattern?; (b) Are there patterns of local adaptation, and how may the species respond to future climate change?; and (c) How many conservation units, which refers to a population of organisms that is considered distinct for purposes of conservation, are present in this species? These investigations provide a new case study on the conservation genomics of threatened species in high‐elevation environments and will shed light on their conservation and management in the face of climate change.

## 2 MATERIALS AND METHODS

### 2.1 Sampling and mRNA sequencing

**FIGURE 1:**
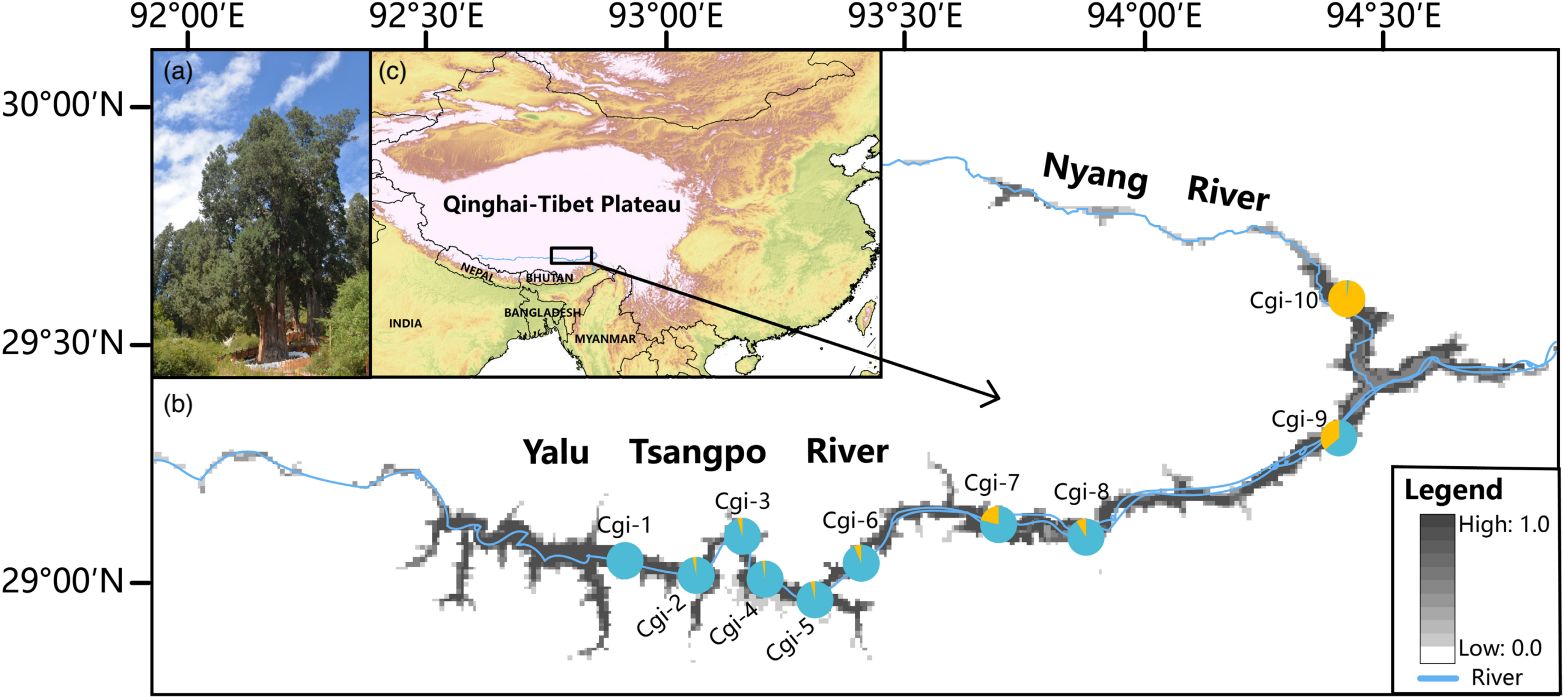
Geographic distribution of sampled *Cupressus gigantea* populations. (a) “The King Cypress”—the largest specimen of *C*. *gigantea* at Nyingchi, is estimated to be more than 3000 years old. (b) The geographic distribution of wild *C*. *gigantea* samples under the background of habitat suitability. (c) The location of the Qinghai–Tibet Plateau. Pie chart shows the ancestral composition of each population with *K* = 2 inferred from ADMIXTURE based on dataset Ⅰ, which contains all SNPs

*Cupressus gigantea*is a narrow endemic conifer occurred in river valleys and hillsides in the middle reaches of the Yalu Tsangpo River and its tributary Niyang River in Southeast Tibet (Figure 1 and Figure S4). We collected fresh needle leaves from 96 *C*. *gigantea* individuals at 10 locations (Figure 1; Table S1). In addition, one individual of *Juniperus microsperma* and one *C*. *funebris* sampled in previous work (Ma et al., 2019) were used as outgroups. Fresh leaves were immediately placed into liquid nitrogen (below −80°C) before RNA extraction. Total RNA was isolated using the TRIzol Reagent (Life Technologies, Thermo Fisher Scientific, Waltham, MA, USA) and the RNeasy Kit (Qiagen, Hilden, Germany) according to the manufacturer's instructions. Prior to RNA fragmentation, poly(A) RNA was purified from total RNA using oligo(dT) magnetic beads. The QIAquick PCR Purification Kit (Qiagen) was used to synthesize and purify cDNA. RNA‐seq libraries were prepared and sequenced on the Illumina HiSeq X Ten platform to generate 150‐bp paired‐end raw reads. TRIMMOMATIC version 0.36 (Bolger et al., 2014) was used to trim and filter the raw reads, discarding adapter sequences, poly‐Ns, and low‐quality reads (*Q* < 30).

### 2.2 Transcriptome *de novo* assembly, read mapping, and SNP calling

Clean reads from one *C*. *gigantea* individual were assembled into contigs using TRINITY version 2.8.5 (Haas et al., 2013) with default settings. Transcripts smaller than 200 bp were discarded, and the longest transcript for each gene was selected for the final assembly. CD‐HIT‐EST version 4.8.1 (Fu et al., 2012) was used to eliminate redundancies and produce unique genes (unigenes) for the final assembly. Subsequently, we removed sequences that were highly similar to contigs in the Microbial Genome Database (MBGD, http://mbgd.genome.ad.jp/; Uchiyama et al., 2010) and to any part of the complete chloroplast genome of *C*. *gigantea* (GenBank accession NC_028155.1; Li et al., 2016). Finally, a reference set of 61,597 unigenes was obtained (Table S3). We used BUSCO version 5.2.2 (Simão et al., 2015) to estimate transcriptome completeness, using the embryophyte conserved gene dataset (embryophyta_odb9) as the query databases, with default settings. TRANSDECODER version 5.5.0 (Haas et al., 2013) was used with default settings to predict the coding regions of the reference transcriptome. After obtaining candidate peptides, BLASTP was used to search for homology to known proteins via the UniProt Knowledgebase (UniProtKB) database, and the human‐readable descriptions for each gene were generated using AHRD version 3.3.3 (Hallab, 2015).

BWA‐MEM version 0.7.17 (Li & Durbin, 2009) was used with default parameters to align high‐quality reads from each *C*. *gigantea* individual and the two outgroup accessions to the reference transcriptome. We used SAMTOOLS version 1.9 (Li et al., 2009) to convert Sequence Alignment/Map (SAM) files to Binary Alignment/Map (BAM) files and to sort the BAM files. PICARD version 2.20.3 (Broad Institute, Cambridge, MA, USA; http://broadinstitute.github.io/picard/) was used to mark PCR duplicates. The RealignerTargetCreator and IndelRealigner tools in GATK version 3.7 (DePristo et al., 2011) were used to realign the regions around indels. Next, SNP calling was performed using the “mpileup” utility in SAMTOOLS version 1.9 with the parameters “‐q 20 ‐Q 20 ‐t AD,ADF,ADR,DP,SP.” (The parameter “AD,ADF,ADR,DP,SP” is interpreted as follows: “AD”: allelic depth, “ADF”: allelic depths on the forward strand, “ADR”: allelic depths on the reverse strand, “DP”: number of high‐quality bases, “SP”: Phred‐scaled strand bias *p*‐value.) Finally, SNPs within 5 bp from any indel were removed.

To satisfy the requirements of various analyses, three SNP datasets were created: a complete dataset (I), and subsets containing only putatively neutral (II) and adaptive (III) loci. For dataset Ⅰ (the complete dataset), SNPs with >50% missing data and a minor allele frequency (MAF) < 0.05 were removed using VCFTOOLS version 0.1.13 (Danecek et al., 2011). In addition, since conifer genomes are known to harbor complex architectures with a high proportion of repeated elements and paralogous genes, we used the program HDPlot (McKinney et al., 2017) to identify and remove putatively paralogous loci.

We used a perl script (Ru et al., 2018) to generate concatenated sequences for each individual, and only fourfold degenerate sites (4DTv) from dataset I were kept. Simultaneously, we used four methods to detect outlier SNPs based on dataset I. Two are *F*_ST_‐based methods: BAYESCAN version 2.1 (Beaumont & Balding, 2004; Foll & Gaggiotti, 2008) and R package PCADAPT version 4.3.3 (Luu et al., 2017; Oksanen et al., 2013). For BAYESCAN, we used 100 prior odds for the model, including 20 pilot runs, followed by 5000 iterations with an initial burn‐in of 50,000 iterations. To minimize false positives, we set the false discovery rate (FDR) to 0.05. For PCADAPT, outlier SNPs were detected with FDR < 0.05 using the QVALUE package in R (Dabney et al., 2010) based on *K* = 2. Another two are genotype–environment association (GEA) approaches: BAYESCENV (De Villemereuil & Gaggiotti, 2015) and a partial canonical redundancy analysis (RDA, (Legendre & Legendre, 2012)) using the R package VEGAN version 2.5.6 (Oksanen et al., 2013; see Section 7 for details). To create the putatively neutral dataset (dataset II), we excluded any outliers detected by BAYESCAN, PCADAPT, BAYESCENV or RDA. Based on all these analyses, dataset Ⅲ (the putatively adaptive dataset) contained the outliers detected by BAYESCAN, PCADAPT, BAYESCENV, or RDA. Dataset II was the 4DTv and excluded any outliers in the dataset Ⅲ based on dataset I.

### 2.3 Environmental data

A total of 19 bioclimatic variables (Table S2) were retrieved for current (1960–1990) and future (2100) periods at 2.5‐min resolution from the WorldClim database (https://www.worldclim.org/; Hijmans et al., 2005). For the future episodes, we calibrated and downscaled climatic projections representing two different future scenarios, SSP‐126 and SSP‐585, from the Sixth Phase of the Coupled Model Intercomparison Project (CMIP6) future climate projections. These reflected moderate and extreme conditions for 2100. All selected environmental layers were converted to the same resolution at grid cell size of 30″ × 30″ using ARCGIS version 10.7 (Environmental Systems Research Institute, Redlands, CA, http://www.esri.com). To avoid multicollinearity, we estimated correlation coefficients between each possible factor combination, then eliminated any factor that had a correlation coefficient greater than |0.8| with two or more other factors by calculating Pearson's correlation coefficient in R. We then randomly eliminated one out of each pair of correlated factors, ultimately retaining a subset of 4 uncorrelated BIOCLIM variables for further analysis: BIO3 (Isothermality), BIO5 (Max Temperature of the Warmest Month), BIO11 (Mean Temperature of the Coldest Quarter), and BIO15 (Precipitation Seasonality).

### 2.4 Genetic structure and phylogenetic inference

To detect evolutionary subdivisions of *C*. *gigantea* populations, we performed ADMIXTURE analysis based on the whole and putatively neutral datasets. The principal component analysis (PCA) was performed using all three datasets (Ⅰ‐Ⅲ). First, PLINK version 1.9 (Chang et al., 2015; Purcell et al., 2007) was used with the parameter “‐‐indep‐pairwise 50 5 0.4” to convert the input format to binary “ped” format and remove linkage disequilibrium sites. ADMIXTURE version 1.3.0 (Alexander & Lange, 2011) was used to estimate the population structure with *K* set to 1–8. The most likely value of *K* was determined by minimizing the cross‐validation error. In addition, PCA was performed using the smartpca program in EIGENSOFT version 6.1.4 (Price et al., 2006).

We conducted phylogenetic analysis using a subset of dataset II from which linkage disequilibrium sites had been removed. A maximum‐likelihood (ML) phylogenetic tree with 200 bootstrap replicates was constructed in RAxML version 8.2.9 (Stamatakis, 2014) with the GTR + GAMMA substitution model using *J*. *microsperma* and *C*. *funebris* as outgroups.

### 2.5 Indices of genetic diversity and population differentiation

Common measures of genetic diversity and differentiation were calculated for all datasets, considering only those eight populations with ≥5 individuals (Table S1). The nucleotide diversity (*π*) was calculated in VCFTOOLS version 0.1.13. Observed heterozygosity (*H*_O_), expected heterozygosity (*H*_E_), and the inbreeding coefficient (*F*_IS_) for each population were calculated using the R package HIERFSTAT version 0.5‐7 (Goudet et al., 2015). Percentage polymorphism (%*Poly*) was calculated in ARLEQUIN version 3.5.2 (Excoffier & Lischer, 2010). The number of private alleles (*Ap*) was calculated in the R package POPPR version 2.9.3 (Kamvar et al., 2014). Pairwise *F*_ST_ between populations was calculated using VCFTOOLS version 0.1.13.

### 2.6 Demographic history

The FASTSIMCOAL2 program (FSC2; Excoffier et al., 2013) was used to infer divergence times and gene flow between *C*. *gigantea* lineages. We also used 4DTv sites for demographic inference to reduce the impact of natural selection. SNPs without MAF filtering were filtered to remove all missing data across all *C*. *gigantea* individuals. Furthermore, VCFTOOLS version 0.1.13 was used to exclude from all downstream analyses those SNPs that deviated (*p* < 0.001) from the Hardy–Weinberg equilibrium from all downstream analyses. We used the perl script *vcf2maf*.*pl* (https://github.com/wk8910/bio_tools/) to construct two‐dimensional joint site frequency spectra (2D‐SFS). Then, eight different evolutionary models (Figure S1) were designed and simulated in FSC2 based on the lineage delineation according to genetic structure. The Akaike information criterion (AIC) was used to determine the best model. Based on previous studies of *Cupressus* species (Ma et al., 2019), we set the average generation time to 50 years and the mutation rate to 7.0 × 10^−9^ per site per generation. Under the condition of 50 independent runs for each bootstrap, we used a parametric bootstrapping approach to construct 95% confidence intervals. In addition, we performed Stairway Plot analysis (Liu & Fu, 2015) to investigate how effective population size changed over time using the folded 1D‐SFS from 4DTv sequences.

### 2.7 Genomic signatures of selection and local adaptation

Two methods were used to measure the associations between all SNPs and climatic gradients: BAYESCENV (De Villemereuil & Gaggiotti, 2015) and redundancy analysis (RDA; Legendre & Legendre, 2012), representing univariate and multivariate GEA approaches, respectively. We used BAYESCENV to test for the sensitivity of each locus to environmental differentiation, using 20 pilot runs with 50,000 MCMC iterations each and an initial burn‐in of 50,000 iterations for each of the four environmental parameters. All other options were kept at the default settings.

RDA was used to explore associations between multilocus SNP. RDA is an analogue of multiple regression that allows simultaneous analysis of multivariate response data (Forester et al., 2018). Linear combinations of response variables (SNP loci) are referred to as RDA canonical axes, any of which are potentially causative, and each RDA axis is correlated with multiple predictor variables (climate variables).

Redundancy analysis and associated analyses were implemented in R using the VEGAN package version 2.5.6 (Oksanen et al., 2013). The four WorldClim climatic variables described above were included as explanatory variables in the analysis. We also checked for multicollinearity using variance inflation factors (VIFs). The value of VIF was <10 for all variables, and therefore, all variables were included in further analysis (James et al., 2013). In addition, RDA requires that the dataset does not have any missing data (i.e., missing SNP genotypes), so each gap in the data was filled with the most common genotype for that locus across individuals (Forester et al., 2018; Harrisson et al., 2017). We used the function “anova.cca” from the VEGAN package (999 permutations) to check the significance of our model through an ANOVA‐like permutation test, and all significant axes were considered to calculate outliers. Then, we determined which SNPs were candidates for local adaptation by identifying those present in the tails of a ±2.5 SD cutoff (two‐tailed *p*‐value = 0.012) on the SNP loadings to identify candidate SNPs (Carvalho et al., 2021). To identify the biological processes associated with the genes identified as candidates for GEA outliers, we performed a Gene Ontology (GO) enrichment analysis using the Bioconductor package TOPGO version 2.24.0 (Alexa & Rahnenführer, 2016).

### 2.8 Prediction of genomic vulnerability to future climate change

To describe the effect of environmental gradients more accurately on genetic variation and to identify spatial areas where gene–environment relationships are most likely to be disrupted by future climate changes, we used the gradient forest (GF) model to predict genetic compositions under future conditions using the R package GRADIENTFOREST (Ellis et al., 2012). GF is a flexible model that uses a machine‐learning regression tree approach to directly model the compositional turnover in genomic variation and efficiently accommodate nonlinear gene–environment relationships (Ellis et al., 2012; Fitzpatrick & Keller, 2015). We included only populations with ≥5 individuals to ensure robust regression, and we executed the GF model using the four uncorrelated BIOCLIM variables and the Moran's eigenvector map (MEM) (Borcard & Legendre, 2002; Dray et al., 2006), which represents the effects of spatial processes and unmeasured environmental variation. The MEM variables were calculated in the R package SPACEMAKER (Dray, 2013), and the SNP data were converted into minor allele frequencies per population. We used 500 regression trees per SNP to fit each GF model using default settings. Because false positives may be induced by rare alleles, we only included SNPs with MAF >10%. Also, to ameliorate some of the problems that arise from linkage, we retained only one SNP per contig for GF analyses. To visualize the results of the GF model across the entire distribution of *C*. *gigantea*, we created 10,000 random points and harvested BIOCLIM and MEM values.

“Genomic vulnerability,” termed “genetic offset” by Fitzpatrick and Keller (2015), is a measure of the mismatch between genotype and the future environment based on the current climate gradient. We extended the GF model to predict genomic vulnerability following Bay et al. (2018) and Fitzpatrick and Keller (2015). We evaluated the predicted genomic composition in 2100 under two shared socioeconomic pathway (SSP) scenarios, SSP‐126 and SSP‐585, which represent low and high greenhouse gas emission trajectories, respectively. We then calculated the Euclidian distance between current and future genetic compositions to identify genomic vulnerability, with the assumption that this correlates positively with the vulnerability of the population in the future. Finally, we mapped the Euclidian distance in geographic space to illustrate the distribution of *C*. *gigantea* populations.

## 3 RESULTS

### 3.1 Reference transcriptome assembly and SNP calling

The assembled reference transcriptome contained 61,597 unigenes with an average length of 904 bp and a contig N50 of 1837 bp (Table S3). The result of BUSCO quality assessments was 84.90%. A total of 26,072 unigenes were annotated using the Swiss‐Prot protein sequence database. After two *F*_ST_‐based methods, we found that PCADAPT identified 653 SNPs and BAYESCAN found 520 outlier SNPs. Two GEA methods identified 2833 outlier SNPs, 611 SNPs for BAYESCENV and 1759 SNPs for RDA (Figure S2). Under our parameter settings, three SNP datasets were obtained: (a) dataset Ⅰ (all SNPs, 145,336 SNPs), (b) dataset Ⅱ (putatively neutral dataset, 26,103 SNPs), and (c) dataset Ⅲ (putatively adaptive dataset, 2833 SNPs).

### 3.2 Population structure and phylogenetic hypothesis

**FIGURE 2:**
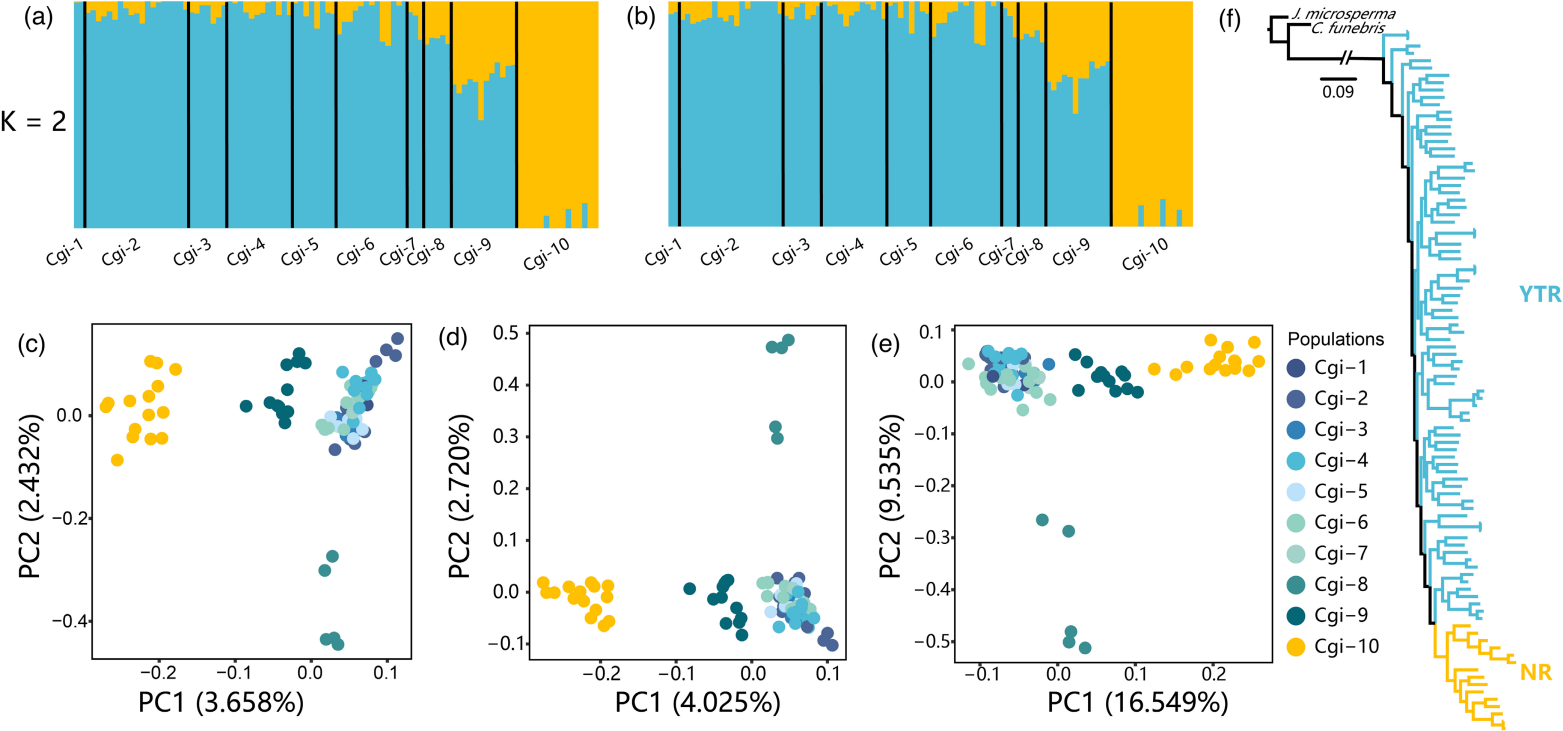
Population structure and phylogenetic inference of *Cupressus gigantea*. (a–b) Structure results, with different colors indicating different genetic backgrounds based on dataset Ⅰ (a) and dataset Ⅱ (b). (c–e) Results of the principal component analysis using dataset Ⅰ (c), dataset Ⅱ (d), and dataset Ⅲ (e). (f) A maximum‐likelihood phylogenetic tree based on 4DTv sites using dataset Ⅰ

ADMIXTURE analyses based on all SNPs (dataset I) and putatively neutral SNPs (dataset II) indicated that *K* = 2 (CV value = 0.54478 and 0.54674, respectively) was optimal (Figure 2a–b; Figure S3). In scenarios based on dataset I or II, Cgi‐10 was distinct from all other populations when *K* = 2. Therefore, there were two clear genetic lineages: the NR lineage comprising Cgi‐10 alone and the YTR lineage comprising all other populations.

The principal component analysis (PCA) revealed patterns similar to those obtained with ADMIXTURE. From datasets I and II, the first principal component (PC1, variance explained = 3.658% and 4.025%, respectively) clearly separated Cgi‐10 (NR lineage) from all other populations, although among the remaining populations (YTR lineage), Cgi‐9 was slightly separated from all others and approached Cgi‐10 slightly along PC1 (Figure 2c,d). In both datasets I and II, PC2 (2.432% and 2.720%, respectively) strongly separated Cgi‐8 from all other populations (Figure 2c,d). Within dataset III, both the PC1 and PC2 axes strongly separated Cgi‐8 from all other populations and also separated Cgi‐10 from populations 1–7, with Cgi‐9 intermediate again (Figure 2e). From all three datasets, populations 1–7 appeared completely intermixed along both axes (Figure 2c,d,e).

In the maximum‐likelihood tree of the ten populations, Cgi‐8 and Cgi‐10 were nested within the YTR lineage, although in each case, all individuals from these populations clustered together (Figure 2f).

### 3.3 Genetic diversity and population differentiation

Measures of genetic diversities had similar trends between dataset Ⅰ and Ⅱ (Table 1 and Table S4). Overall, most YTR populations had higher levels of diversity than the NR lineage (Cgi‐10), and within the YTR lineage, Cgi‐6 and Cgi‐4 had the highest diversity scores, including nucleotide diversity (*π*), observed heterozygosity (*H*_O_), and heterozygosity within populations (*H*_E_). Population Cgi‐8 had the lowest diversity scores across datasets Ⅰ and Ⅱ (Table 1 and Table S4), while in dataset III, it had the higher diversity scores than all other populations except Cgi‐10. Values of the inbreeding coefficient (*F*_IS_) ranged from −0.0283/−0.0392 (Cgi‐6) to −0.2324/−0.2350 (Cgi‐8) for datasets Ⅰ and Ⅱ and ranged from −0.3881 (Cgi‐8) to 0.0491 (Cgi‐2) for dataset Ⅲ (Table S4), indicating relatively low inbreeding in *C*. *gigantea*. The percentage polymorphism (%*Poly*) varied from 51.80/58.37% in Cgi‐9 to 62.58/68.11% in Cgi‐3 for datasets Ⅰ and Ⅱ and varied from 21.12% (Cgi‐2) to 55.91% (Cgi‐8) for dataset Ⅲ.

**TABLE 1:** Measures of diversity and pairwise *F*_ST_ for 96 *Cupressus gigantea* individuals from dataset Ⅰ

Lineage	Population	*N*	*π*	*H*_O_	*H*_E_	*F*_IS_	*%Poly*	*Ap*	Pairwise *F*_ST_
YTR	Cgi‐2	19	0.0026	0.3321	0.3267	−0.0381	57.28	18	0.0573
	Cgi‐3	7	0.0026	0.3249	0.3221	−0.0878	62.58	0	0.0647
	Cgi‐4	12	0.0027	0.3339	0.3326	−0.0472	59.09	0	0.0600
	Cgi‐5	8	0.0025	0.3296	0.3176	−0.1016	62.08	0	0.0669
	Cgi‐6	13	0.0054	0.3310	0.3321	−0.0283	54.88	0	0.0559
	Cgi‐8	5	0.0022	0.3288	0.2865	−0.2324	59.11	0	0.1024
	Cgi‐9	12	0.0051	0.3359	0.3225	−0.0704	51.80	0	0.0502
	All (comparing to NR)	81	0.0054	0.3332	0.3333	0.0076	45.90	18	0.0488
NR	Cgi‐10	15	0.0049	0.3079	0.2997	−0.0515	57.75	102	–
	All (comparing to YTR)	15	0.0049	0.3079	0.2997	−0.0515	57.75	102	–
ALL	‐	96	0.0027	0.3313	0.3348	0.0194	43.57	–	–

Note

In each column, warmer colors reflect higher values. *N*, number of individuals in the population; *π*, nucleotide diversity (calculated within variant loci); *H*_O_, observed heterozygosity; *H*_E_, heterozygosity within populations; *F*_IS_, inbreeding coefficient; *%Poly*, percentage of polymorphism; *Ap*, the number of private alleles; and Pairwise *F*_ST_, mean Weir and Cockerham's (1984) pairwise *F*_ST_ relative to the NR lineage.

The *F*_ST_ values between the YTR and NR lineages were 0.0488/0.0474/0.2199 for datasets Ⅰ–Ⅲ (Table 1 and Tables S4–S6). Cgi‐10 and Cgi‐8 had higher values of pairwise *F*_ST_ (*F*_ST_ = 0.1024/0.1017/0.3361), whereas Cgi‐10 and Cgi‐9 had the lowest *F*_ST_ (*F*_ST_ = 0.0502/0.0494/0.1444). In general, dataset III produced the highest *F*_ST_ values.

### 3.4 Gene flow and population demography

**FIGURE 3:**
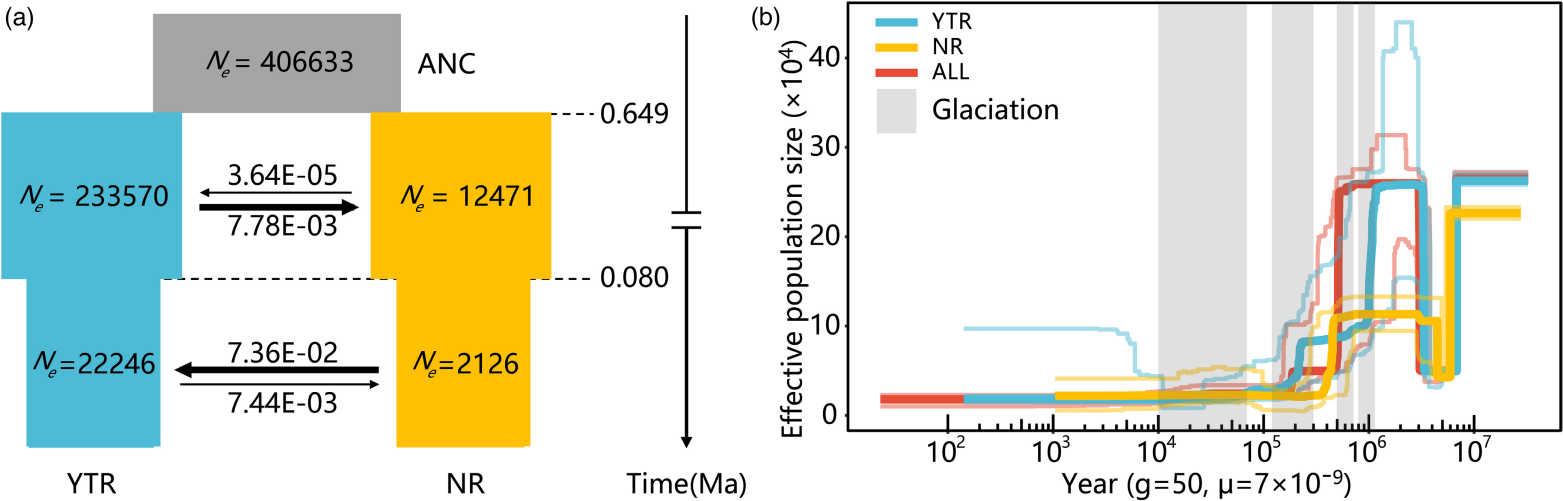
Demographic history of *Cupressus gigantea*. (a) Schematic diagram of demographic scenario modeled in FSC2. Estimated effective population sizes and divergence times are indicated. The numbers next to the arrows indicate the per generation migration rate between populations. (b) The detailed population demographic history of YTR, NR, and all *C*. *gigantea* individuals (ALL) over the past 10 million years based on the Stairway Plot method. The inferred effective size *N_e_* of the *C*. *gigantea* population is plotted from present time (0) to the past. Thick lines represent the median, and thin light lines represent the 95% pseudo‐CI defined by the 2.5% and 97.5% estimates from the SFS analysis. The periods of the Xixiabangma Glaciation, the Naynayxungla Glaciation, the Guxiang Glaciation, and the Baiyu Glaciation are highlighted with gray vertical bars

We simulated eight candidate migration models to investigate the demographic history of *C*. *gigantea* based on the results described above. The best‐fitting model showed that the divergence between the NR and YTR lineages occurred approximately 0.649 million years ago (Ma) (Figure 3a; see Tables S7 and S8 for relative likelihood of candidate models, such as AIC values, and estimated demographic parameters of each model), and both lineages experienced sharp contractions in population size around 0.08 Ma. The estimated ancient gene flow was consistently higher from the YTR to the NR lineage than in the opposite direction, especially before 0.08 Ma when it was ca. 214 times higher, dropping to ~9 times lower between 0.08 Ma and the present.

We performed a Stairway Plot analysis for all SNPs based on dataset I and for the NR and YTR lineages alone. This analysis revealed slightly different demographic histories between YTR and NR (Figure 3b). All populations experienced a sharp decline in population size around 7 Ma, with recovery occurring ~4.5 Ma for NR and ~3 Ma for YTR and for the species as a whole (Figure 3b). The YTR lineage then experienced two further reductions in population size of about 1.2–0.8 Ma and 0.24–0.2 Ma, coinciding with the timing of the Xixiabangma and Guxiang Glaciations, respectively. The NR lineage experienced a sharp population contraction around 0.5 Ma at the end of the Naynayxungla Glaciation, after which it maintained an extremely small population size.

### 3.5 Genomic signatures of selection and local adaptation

Based on all SNPs, both BAYESCENV and RDA indicated a number of candidate loci with significant genotype‐by‐environment associations. BAYESCENV identified 611 (0.42% of 145,336 SNPs) outlier SNPs with *q* values <0.05. The most SNP associations were found for BIO15 (Precipitation Seasonality, 289 SNPs), followed by BIO5 (Max Temperature of the Warmest Month, 250 SNPs), BIO11 (Mean Temperature of the Coldest Quarter, 182 SNPs), and BIO3 (Isothermality, 174 SNPs; Table S9). RDA detected 1,759 (1.21%) significant associations between the environmental variables and the SNPs. One thousand and twelve SNPs (58.04%) were correlated most to BIO15, 627 SNPs (35.64%) most to BIO3, 67 SNPs (3.81%) most to BIO5, and 44 SNPs (2.51%) most to BIO11 in the RDA (Table S9). Combining the two methods, there were 60 outlier SNPs identified by both BAYESCENV and RDA, and 2310 outlier SNPs identified in total by either of the two methods. To address the potential functional relevance of the environment‐associated outlier genes, we conducted a functional annotation of the predicted gene sequences in the *C*. *gigantea* transcriptome reference. Among the 2310 environment‐associated outliers, GO categories could be assigned to 205 genes. Among these, we found that there were 28 categories to be significantly overrepresented (*p* < 0.01; Table S10). These categories represented a broad range of biological processes, such as stress response, metabolism, and ion and protein transport.

### 3.6 Spatial distribution of gene–environment associations and future genomic mismatch

**FIGURE 4:**
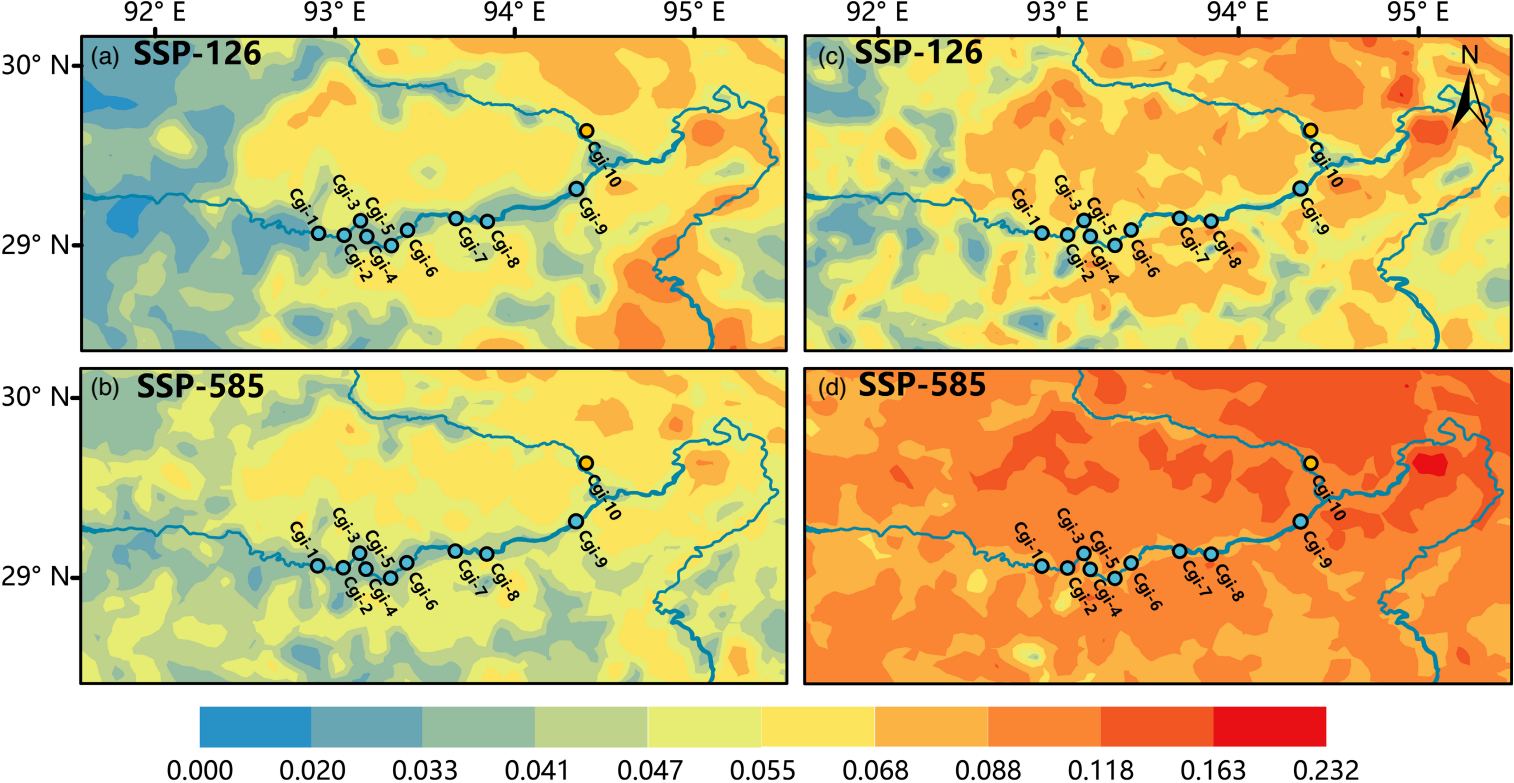
Prediction of genomic mismatch under future climate change for two shared socioeconomic pathways (SSPs) in 2100 based on the whole SNPs (a, b) and environment‐associated SNPs (c, d). Red and blue indicate high and low genomic mismatch, respectively. The circles represent the locations of *Cupressus gigantea* populations used in our study

We simulated the genomic change needed to track predicted climate change up until the year 2100 under two greenhouse gas scenarios (SSP‐126 and SSP‐585; Figure 4). We found that *C*. *gigantea* growing along the river bank had a lower genetic offset, but individuals that grew in the valley far from the bank suffered from higher genomic vulnerability, indicating that selection pressure due to climate would be higher. Moreover, the NR lineage had a higher genetic offset compared with YTR populations, indicating that individuals in the NR lineage may face more severe environmental selection pressure. Compared with all SNPs, we found that those showing adaptive variation were more susceptible to strong climate change. Finally, as the predicted climate becomes increasingly warm, the potential suitable habitat for *C*. *gigantea* became more threatened, and in the most extreme case (SSP‐585 2100), there was a high level of genomic vulnerability across almost the entire distribution range (Figure 4d).

## 4 DISCUSSION

We used up to 145,336 SNPs derived from next‐generation sequencing to survey the genetic diversity, population structure, demographic history, and potential response to future climate change of *C*. *gigantea*. This rare conifer occurs in the Yalu Tsangpo River valley (Figure S4) and is well known for its largest individual, the King Cypress. We found that the species' populations can be divided into two lineages, the YTR and NR lineages, each of which corresponds to an independent management unit (*MU*). The former comprises nine of the ten known populations, occupies a much wider distribution range, and exhibits higher genetic diversity. Based on current genetic–environmental associations and predictive climate modeling, we identified regions with high genetic offset in the *C*. *gigantea* distribution range and found that the population containing the King Cypress (Cgi‐10, NR) will be the most vulnerable to future climate change.

### 4.1 Demographic history and intraspecific divergence of *Cupressus gigantea*

Our analysis of demographic history using FSC2 revealed the divergence history of *C*. *gigantea* populations (Figure 3a). The divergence between YTR and NR occurred 0.649 Ma, coinciding with the Naynayxungla Glaciation (0.72–0.50 Ma) on the Qinghai–Tibet Plateau *s*.*l*. (Zheng et al., 2002). Thus, the genetic differentiation between YTR and NR lineages likely reflects regional climate fluctuations, which may have generated a gene flow barrier between them. Similar patterns of intraspecific differentiation have been observed in other Qinghai–Tibet Plateau endemic species, such as *C*. *chengiana* (Li, et al., 2020, 2021, *Aconitum gymnandrum* (Wang et al., 2009), and *Eospalax baileyi* (Tang et al., 2010).

Meanwhile, *C*. *gigantea* experienced a demographic history that included multiple bottleneck events. According to the Stairway Plot (Figure 3b), *C*. *gigantea* populations experienced three bottlenecks that occurred at about 7, 0.5, and 0.2 Ma. The periods of effective population size (*N_e_*) reduction preceding these coincided respectively with the decline in atmospheric surface air temperature during the late Miocene (Sun & An, 2005), the development of the Naynayxungla Glaciation (0.72–0.50 Ma), and the Guxiang Glaciation (ca. 0.30–0.13 Ma; Zheng et al., 2002). This suggests that the periods of population contractions in *C*. *gigantea* were associated with paleoclimatic changes during the Miocene and Pleistocene. Nevertheless, it is important to bear in mind that the accuracy of Stairway Plot analysis (Figure 3b) in detecting bottlenecks may decay at deeper evolutionary timescales (Liu & Fu, 2015). The slightly different demographic trajectories of YTR and NR may be attributed to the effects of interacting factors such as ecology, local topography, and geological history on the species' response to climate change (Avise, 2000; Hewitt, 1996, 2000, 2004; Scheffers et al., 2016). However, consistent with Ma et al. (2019), the effective population sizes of both YTR and NR lineages have declined to relatively low levels compared with those of other wind‐pollinated tree species, causing a higher relative risk of extinction in *C*. *gigantea* (Hutchings, 2015). Another hypothesis for the reduction in effective population size is that low genetic diversity of *C*. *gigantea* (Table 1, Tables S4 and S5) may have reduced the species' ability to adapt and survive during short‐ and long‐term environmental changes (Allendorf et al., 2010; Ellstrand & Elam, 1993; Hamilton, 2011; Lande et al., 1999).

### 4.2 Local adaptation of *Cupressus gigantea* populations

In addition to the history of divergence and demographic changes, environmental factors might have also contributed to the current genomic structure of *C*. *gigantea*. We used both univariate and multivariate methods to detect the selection characteristics related to climate change across the range of *C*. *gigantea*. Significant environment‐associated SNPs were detected in candidate genes chosen *a priori* based on broad functional categories, and also in random fragments with retrieved functional annotations (Table S10). We potentially identified 2310 candidate loci under selection (detected by BAYESCENV or RDA), of which 205 are within functional genes annotated in the *C*. *gigantea* transcriptome. Climate variables related to temperature and precipitation limit the survival, growth, and productivity of many forest tree species (Peterson & Peterson, 2001). Of the climate variables included in the BAYESCENV, Precipitation Seasonality (32.29%) and Max Temperature of the Warmest Month (27.93%) explained the most variance in SNP genotypes. The multivariate gene association methods identified 1759 outlier SNPs, of which 58.04% were related to Precipitation Seasonality. The GO enrichment analyses suggested that these outliers were significantly (*p* < 0.01) enriched in many categories related to stress responses, such as response to singlet oxygen (GO: 0000304), reactive oxygen species (GO: 0000302), hypoxia (GO: 0001666), and light stimulus (GO: 0009416). In addition, ion transport (GO: 0006811), tissue development (GO: 0009888), MAPK cascade (GO: 0000165), and ethylene biosynthetic process (GO: 0009693) were also enriched, which might have contributed to abiotic stress responses and phytohormone treatments. However, we emphasize that our study is only able to detect loci potentially associated with climate variables, while the actual agents and targets of selection need to be rigorously established through manipulation experiments.

### 4.3 Climate‐driven genomic vulnerability

Understanding the genetic basis of adaptation to climate change remains still a very important task (Capblancq et al., 2020). Based on current genetic–environmental associations, we attempted to assess which populations would be most vulnerable to future climate change. We used the method of Fitzpatrick and Keller (2015), which has been applied to a variety of species (Bay et al., 2018; Vranken et al., 2021; Zhao et al., 2020), to calculate the mismatch between genotypes and future predicted environments using an extension of the gradient forest (GF) analysis. The populations with the highest degree of mismatch are least likely to adapt rapidly to climate change, and this may lead to decline or extinction (Bay et al., 2018).

Further GF analysis revealed a clear signal of potential risk for *C*. *gigantea* populations under global climate change, especially with regard to adaptive variation (Figure 4). The genetic mismatch is much higher in the northeastern populations, particularly Cgi‐10 (the NR population), which have relatively lower genetic diversity and the highest differentiation relative to the other populations. Populations in these high‐risk regions will need to adapt more quickly to keep pace with the rapidly changing climate. However, like other conifers, *C*. *gigantea* has a long lifespan and generation time; these characteristics often lead to slower adaptation in response to rapid climate changes (Zhao et al., 2020). Because the Cgi‐10 population has experienced severe bottlenecks that have caused a very small effective population size, and given the low mutation rate in conifers (De La Torre et al., 2017), persistence of this population *in situ* in the context of global climate change seems a formidable challenge. The largest and most famous *C*. *gigantea* individual, the King Cypress, is located in Cgi‐10, and future climate change appears to pose a severe threat to the entire population and this specific individual.

The genomic vulnerability predicted here for *C*. *gigantea* is greater than that of the widely distributed *Platycladus orientalis* (Jia et al., 2020) and *Pinus densata* (Zhao et al., 2020), and indicates that climate change will likely exceed its adaptive capacity. Jia et al. (2020) showed that *P*. *orientalis* populations in the southern and northern margins would face more severe challenges. By contrast, the mismatch is higher in western *P*. *densata* populations where differentiation (relative to other populations) is highest and genetic diversity lowest according to Zhao et al. (2020). Our results showed that within its distribution range, areas far away from river channels were not suitable for *C*. *gigantea* survival, meaning that the species will most likely survive only in river channels. On a whole, *in situ* persistence of many *C*. *gigantea* populations will be a significant challenge under future climate regimes, especially for Cgi‐10.

However, although our results may provide new insight into how *C*. *gigantea* will respond to future climate changes, we would like to warn the readers that genomic predictions of climate (mal)adaptation, as we have conducted here, are still in their infancy and face numerous challenges and uncertainties that await further investigation (Capblancq et al., 2020).

### 4.4 Delineation of conservation units and conservation implications

Given the importance of genetic diversity for natural populations, especially for species that are already endangered or patchily distributed with limited dispersal ability, one of the most important current challenges is maximizing the species' evolutionary potential by delineating appropriate conservation units (*CU*s) before developing conservation strategies (Barbosa et al., 2018; Funk et al., 2012; Liu et al., 2020). The characterization of *CU*s can now benefit from greater information on species' natural and adaptive variation revealed by genomic data, and further categories are recognized: (a) evolutionarily significant units (*ESU*s), which are monophyletic (all individuals share a common ancestor) lineages that have experienced independent evolutionary history from, but no recent gene flow with, other *ESU*s; (b) management units (*MU*s), that is, groups of populations that are demographically independent; and (c) adaptive units (*AU*s), which reflect adaptive differentiation related to landscape and climate (Barbosa et al., 2018; Funk et al., 2012).

In the present study, similar patterns were detected based on all SNPs and on putatively neutral SNPs, whereas the results from the dataset of candidate positively selected SNPs were different. Based on the putatively neutral dataset, ADMIXTURE, PCA, and phylogenetic analyses revealed the existence of two genetic lineages (NR and YTR) in *C*. *gigantea* (Figure 2), corresponding to populations Cgi‐10 from the Nyang River valley, and nine populations from Yalu Tsangpo River valley, respectively. In addition, coalescent analysis suggested that the two lineages have different demographic histories. All these lines of evidence suggest that there are two *MU*s, the YTR and the NR (Barbosa et al., 2018; de Guia & Saitoh, 2007; Funk et al., 2012). Hence, two *CU*s can be recognized, but we caution that our identification of NR *MU* is based on a limited sampling size (Table S1), and future population genomic surveys with more samples from these two populations are needed to confirm our findings.

The appropriate delineation of effective *CU*s is the most important basis for successful biological conservation (Li & Ge, 2002), and each of the two *CU*s comprising *C*. *gigantea* faces different conservation issues. We therefore recommend that the threatened status of each *CU* should be assessed independently. According to our genetic–climatic association analysis based on GF models, the northeastern populations have a higher genetic offset, meaning that the NR *MU* is the most vulnerable in the face of climate change. Similarly, Zheng et al. (2007) found that populations of *C*. *gigantea* near the Nyang River are at the stage of population aging and that populations in the area where the Yalu Tsangpo River meets its tributary the Nyang River are also gradually shrinking in size. In view of these observations, we believe that the NR *MU* should be considered as a higher priority *CU*. Compared with other populations, the YTR *MU* is relatively safe, as it has higher genetic diversity, lower population differentiation, a larger estimated *N_e_*, a lower genetic offset, and a stable age structure (Zheng et al., 2007).

In its native range in southeastern Tibet, *C*. *gigantea* has significant ecological value, but its range has become fragmented and highly restricted near the arid or semiarid valley in the middle reaches of the Yalu Tsangpo River and its tributary the Nyang River. Our study highlights the potential factors that put *C*. *gigantea* populations at risk. Specifically, three bottlenecks caused by intensive historical climate change events have led to the loss of genetic diversity and declines in effective population size. Moreover, Wang et al. (2005) have proposed that the internal factor of low seed vigor and the external factor of severe seed injury by diseases, birds, and mice have increased its vulnerability to extinction. We propose that both *in situ* and *ex situ* conservation approaches should be applied to this species. Each *CU* should be given independent *in situ* conservation and management. In addition, *ex situ* conservation measures such as assisted migration, seedling orchard establishment, and seed storage should be seriously considered for the whole distribution range and all conservation units.

## CONFLICT OF INTEREST

None declared.

## Supporting information

Supplementary Material

## Data Availability

The transcriptome sequencing data have been deposited in National Genomics Data Center (https://bigd.big.ac.cn/) under BioProject: PRJCA006268.
