# Supplementary material for: Genomic insights into the genotype–environment mismatch and conservation units of a Qinghai–Tibet Plateau endemic cypress under climate change
Source: Evol Appl. 2022 Jun 10;15(6):919–33. doi: 10.1111/eva.13377 (PMC9234613; doi:10.1111/eva.13377)
Supplement: Supplementary file 1 — Supplementary Material [file EVA-15-919-s001.docx]

**Methods and Materials**

**Species distribution modeling**

An ensemble approach was used to forecast potential distributions of *C. gigantea* with R package BIOMOD2 (Thuiller, Georges, Engler, & Breiner, 2013). To obtain locations that were not sampled in this study, we searched through the Global Biodiversity Information Facility (GBIF) and published papers (Fu et al., 2019; Wang et al., 2017). In total, we identified 42 *C. gigantea* sites (10 from this study, 17 from the GBIF, and 15 from previous papers). We used ensemble models with three modeling algorithms: generalized linear model (GLM), generalized additive model (GAM), and random forest (RF). Because every SDM required known presence and absence (PA) records, we generated two sets of PAs by selecting 1000 random points across the distribution range of *C. gigantea* (Barbet‐Massin, Jiguet, Albert, & Thuiller, 2012). We randomly selected 70% of the distribution data for modeling and used the remaining 30% for model validation. The area under the receiver operating characteristic curve (AUC; (Fielding & Bell, 1997)) and the true skill statistic (TSS; (Allouche, Tsoar, & Kadmon, 2006)) were used to evaluate the accuracy of the BIOMOD2 prediction models. Those models with TSS > 0.5 were included to build an ensemble model. The calibrated model was then projected to current climatic space.

**Results**

**Potential distribution of *Cupressus gigantea***

We used the ensemble model in BIOMOD2 to perform ecological niche modelling of *C. gigantea*. According to the current model (Figure S3), *C. gigantea* has a very small range of suitable habitats located in the cold temperate zone of the Qinghai-Tibet Plateau (about 29°–30° N, 92°–95° E). The potential geographic distribution of *C. gigantea* predicted in the current model was basically the same as that recorded in iPlant (<http://www.iplant.cn>).

**Reference**

Allouche, O., Tsoar, A., & Kadmon, R. (2006). Assessing the accuracy of species distribution models: prevalence, kappa and the true skill statistic (TSS). *Journal of Applied Ecology*, 43(6), 1223–1232. doi:10.1111/j.1365-2664.2006.01214.x

Barbet-Massin, M., Jiguet, F., Albert, C. H., & Thuiller, W. (2012). Selecting pseudo-absences for species distribution models: how, where and how many? *Methods in Ecology and Evolution*, 3(2), 327–338. doi:10.1111/j.2041-210X.2011.00172.x

Fielding, A. H., & Bell, J. F. (1997). A review of methods for the assessment of prediction errors in conservation presence/absence models. *Environmental Conservation*, 24(1), 38–49. doi:10.1017/S0376892997000088

Fu, Y., Li, S., Guo, Q., Zheng, W., Yang, R., & Li, H. (2019). Genetic diversity and population structure of two endemic *Cupressus* (Cupressaceae) species on the Qinghai-Tibetan plateau. *Journal of Genetics*, 98(1), 14. doi:10.1007/s12041-019-1059-4

Thuiller, W., Georges, D., Engler, R., & Breiner, F. (2013). biomod2: Ensemble platform for species distribution modeling. *R package version*, 2(7), r560.

Wang, Y., Li, Z., Liu, S., Li, S., Zhao, S., Lei, P., … Sang, L. (2017). Analysis of genetic diversity of Cupressus gigantea in Tibet by ISSR marker. *Liaoning Forestry ence and Technology* (6), 12–14.

**TABLE S1** Locations of 10 *Cupressus gigantea* populations.

| **Population** | **Latitude (°N)** | **Longitude(°E)** | **Altitude (m)** | **N** |
| --- | --- | --- | --- | --- |
| Cgi-1 | 29.07 | 92.93 | 3131 | 2 |
| Cgi-2 | 29.05 | 93.06 | 3297 | 19 |
| Cgi-3 | 29.14 | 93.14 | 3159 | 7 |
| Cgi-4 | 29.05 | 93.16 | 3190 | 12 |
| Cgi-5 | 29.00 | 93.31 | 3120 | 8 |
| Cgi-6 | 29.07 | 93.39 | 3059 | 13 |
| Cgi-7 | 29.13 | 93.85 | 3010 | 3 |
| Cgi-8 | 29.16 | 93.67 | 3133 | 5 |
| Cgi-9 | 29.34 | 94.38 | 2959 | 12 |
| Cgi-10 | 29.63 | 94.39 | 3129 | 15 |

**TABLE S2** Environmental variables used in this study.

| **Name** | **Description** |
| --- | --- |
| BIO1 | Annual Mean Temperature |
| BIO2 | Mean Diurnal Range |
| BIO3 | Isothermality |
| BIO4 | Temperature Seasonality |
| BIO5 | Max Temperature of the Warmest Month |
| BIO6 | Min Temperature of the Warmest Month |
| BIO7 | Temperature Annual Range |
| BIO8 | Mean Temperature of the Wettest Quarter |
| BIO9 | Mean Temperature of the Driest Quarter |
| BIO10 | Mean Temperature of the Warmest Quarter |
| BIO11 | Mean Temperature of the Coldest Quarter |
| BIO12 | Annual Precipitation |
| BIO13 | Precipitation of the Wettest Month |
| BIO14 | Precipitation of the Driest Month |
| BIO15 | Precipitation Seasonality |
| BIO16 | Precipitation of the Wettest Quarter |
| BIO17 | Precipitation of the Driest Quarter |
| BIO18 | Precipitation of the Warmest Quarter |
| BIO19 | Precipitation of the Coldest Quarter |

**TABLE S3** Summary of reference transcriptome.

| Number of unigenes | 61597 |
| --- | --- |
| Number of transcripts | 61597 |
| Percent GC | 41.13% |
| Contig N10 (bp) | 4379 |
| Contig N20 (bp) | 3323 |
| Contig N30 (bp) | 2681 |
| Contig N40 (bp) | 2233 |
| Contig N50 (bp) | 1837 |
| Median contig length (bp) | 408 |
| Average contig (bp) | 904.26 |
| Total assembed bases (bp) | 55699983 |
| Complete BUSCOs | 84.90% |

**TABLE S4** Measures of diversity and pairwise *F*_ST_ for 96 *Cupressus gigantea* individuals from dataset Ⅱ.

| **Lineage** | **Population** | **N** | ***H***_O_ | ***H***_E_ | ***F***_IS_ | ***%Poly*** | ***Ap*** | **Pairwise *F*_ST_** |
| --- | --- | --- | --- | --- | --- | --- | --- | --- |
| YTR | Cgi-2 | 19 | 0.3395 | 0.3320 | -0.0421 | 64.49 | 1 | 0.0552 |
|  | Cgi-3 | 7 | 0.3329 | 0.3272 | -0.0923 | 68.11 | 0 | 0.0643 |
|  | Cgi-4 | 12 | 0.3447 | 0.3385 | -0.0576 | 62.47 | 0 | 0.0582 |
|  | Cgi-5 | 8 | 0.3389 | 0.3227 | -0.1092 | 67.19 | 0 | 0.0656 |
|  | Cgi-6 | 13 | 0.3409 | 0.3376 | -0.0392 | 60.47 | 0 | 0.0555 |
|  | Cgi-8 | 5 | 0.3315 | 0.2872 | -0.2350 | 62.30 | 0 | 0.1017 |
|  | Cgi-9 | 12 | 0.3429 | 0.3264 | -0.0762 | 58.37 | 0 | 0.0494 |
|  | All (Comparing to NR) | 81 | 0.3415 | 0.3381 | -0.0021 | 51.83 | 1 | 0.0474 |
| NR | Cgi-10 | 15 | 0.3136 | 0.3034 | -0.0557 | 64.13 | 1 | - |
|  | All (Comparing to YTR) | 15 | 0.3136 | 0.3034 | -0.0557 | 64.13 | 1 | - |
| ALL | - | 96 | 0.3389 | 0.3392 | 0.0097 | 49.56 | - | - |

In each column, warmer colors reflect higher values. N, number of individuals in the population; *H*_O_, observed heterozygosity; *H*_E_, heterozygosity within populations; *F*_IS_, inbreeding coefficient; *%Poly*, percentage of polymorphism; *Ap*, the number of private alleles; Pairwise *F*_ST_, Mean Weir and Cockerham’s (1984) pairwise *F*_ST_ relative to the NR group.

**TABLE S5** Measures of diversity and pairwise *F*_ST_ for 96 *Cupressus gigantea* individuals from dataset Ⅲ.

| **Lineage** | **Population** | **N** | ***H***_O_ | ***H***_E_ | ***F***_IS_ | ***%Poly*** | ***Ap*** | **Pairwise *F*_ST_** |
| --- | --- | --- | --- | --- | --- | --- | --- | --- |
| YTR | Cgi-2 | 19 | 0.1131 | 0.1226 | 0.0491 | 21.12 | 1 | 0.3005 |
|  | Cgi-3 | 7 | 0.1512 | 0.1459 | -0.0573 | 31.55 | 0 | 0.2575 |
|  | Cgi-4 | 12 | 0.1376 | 0.1403 | -0.0076 | 27.72 | 0 | 0.2605 |
|  | Cgi-5 | 8 | 0.1225 | 0.1192 | -0.0347 | 25.00 | 0 | 0.2747 |
|  | Cgi-6 | 13 | 0.1938 | 0.1973 | -0.0099 | 26.84 | 0 | 0.2385 |
|  | Cgi-8 | 5 | 0.3037 | 0.2098 | -0.3881 | 52.91 | 0 | 0.3361 |
|  | Cgi-9 | 12 | 0.2226 | 0.2053 | -0.0807 | 30.97 | 0 | 0.1444 |
|  | All (Comparing to NR) | 81 | 0.1684 | 0.2007 | 0.1037 | 19.61 | 1 | 0.2199 |
| NR | Cgi-10 | 15 | 0.3372 | 0.2779 | -0.1920 | 48.54 | 1 | - |
|  | All (Comparing to YTR) | 15 | 0.3372 | 0.2779 | -0.1920 | 48.54 | 1 | - |
| ALL | - | 96 | 0.1951 | 0.2417 | 0.1718 | 18.93 | - | - |

In each column, warmer colors reflect higher values. N, number of individuals in the population; *H*_O_, observed heterozygosity; *H*_E_, heterozygosity within populations; *F*_IS_, inbreeding coefficient; *%Poly*, percentage of polymorphism; *Ap*, the number of private alleles; Pairwise *F*_ST_, Mean Weir and Cockerham’s (1984) pairwise *F*_ST_ relative to the NR group.

**TABLE S6** Pairwise *F*_ST_ based on dataset Ⅰ–Ⅲ (above diagonal) and geographic distances (km, below diagonal) between *Cupressus gigantea* populations.

| **Populations** | **Cgi-2** | **Cig-3** | **Cgi-4** | **Cgi-5** | **Cgi-6** | **Cgi-8** | **Cgi-9** | **Cgi-10** |
| --- | --- | --- | --- | --- | --- | --- | --- | --- |
|  | **dataset Ⅰ–Ⅲ** | **dataset Ⅰ–Ⅲ** | **dataset Ⅰ–Ⅲ** | **dataset Ⅰ–Ⅲ** | **dataset Ⅰ–Ⅲ** | **dataset Ⅰ–Ⅲ** | **dataset Ⅰ–Ⅲ** | **dataset Ⅰ–Ⅲ** |
| Cgi-2 | - | 0.0103/0.0096/0.0663 | 0.0079/0.0073/0.450 | 0.0176/0.0171/0.0828 | 0.0114/0.0109/0.0599 | 0.0640/0.0619/0.4555 | 0.0310/0.0298/0.1938 | 0.0573/0.0552/0.3005 |
| Cig-3 | 11985.53 | - | 0.124/0.0126/0.0581 | 0.0221/0.0222/0.0822 | 0.0138/0.0145/0.0478 | 0.0765/0.0756/0.3633 | 0.0340/0.0337/0.1151 | 0.0647/0.0643/0.2575 |
| Cgi-4 | 9277.28 | 9859.72 | - | 0.0206/0.0208/0.0705 | 0.0059/0.0054/0.0457 | 0.0665/0.0660/0.3677 | 0.0282/0.0271/0.1511 | 0.0600/0.0582/0.2605 |
| Cgi-5 | 24222.61 | 21855.75 | 15208.00 | - | 0.0167/0.0169/0.0466 | 0.0818/0.0803/0.4261 | 0.0386/0.0378/0.1515 | 0.0669/0.0656/0.2747 |
| Cgi-6 | 31706.10 | 25139.73 | 22507.42 | 11115.06 | - | 0.0588/0.0591/0.3265 | 0.0262/0.0264/0.1221 | 0.0559/0.0555/0.2385 |
| Cgi-8 | 59597.24 | 50818.31 | 50673.64 | 39114.15 | 28595.44 | - | 0.0787/0.0782/0.2728 | 0.1024/0.1017/0.3361 |
| Cgi-9 | 131679.78 | 122191.77 | 122857.71 | 110915.01 | 100752.73 | 72211.02 | - | 0.0502/0.0494/0.1444 |
| Cgi-10 | 143714.46 | 132826.02 | 135737.13 | 126282.83 | 115321.46 | 87578.59 | 31950.16 | - |

**TABLE S7** Relative likelihood of the candidate models.

| **Model ID** | **Max(log_10_(Lhoodi))^a^** | **No. of parameters (d)** | **AIC_i_** | **Δ_i_** | **Model normalized relative likelihood (w_i_)** |
| --- | --- | --- | --- | --- | --- |
| Model 1 | −100772.69 | 6 | 464087.40 | 3461.29 | 0.00E+00 |
| Model 2 | −100074.77 | 10 | 460881.35 | 255.23 | 3.7737E−56 |
| Model 3 | −100135.85 | 10 | 461162.62 | 536.50 | 3.1606E−117 |
| Model 4 | −100028.72 | 12 | 460673.28 | 47.17 | 5.73031E−11 |
| Model 5 | −100138.79 | 8 | 461172.17 | 546.06 | 2.6568E−119 |
| Model 6 | −100034.62 | 14 | 460704.45 | 78.34 | 9.76313E−18 |
| Model 7 | −100110.95 | 14 | 461055.96 | 429.85 | 4.56656E−94 |
| Model 8 | −100016.74 | 16 | 460626.11 | 0.00 | 1.00E+00 |

**TABLE S8** Parameters of eight candidate models inferred from FASTSIMCOAL2.

| **Model** | **Scenario** | **NA** | **N_NR_** | **N_YTR_** | **NA_NR_** | **NA_YTR_** | **M_NR_→M_YTR_** | **M_YTR_→M_NR_** | **MA_NR_→MA_YTR_** | **MA_YTR_→MA_NR_** | **T1** | **T2** |
| --- | --- | --- | --- | --- | --- | --- | --- | --- | --- | --- | --- | --- |
| Model 1 | A | 254168 | 8644 | 30088 | - | - | - | - | - | - | 50900 |  |
| Model 2 | B | 458021 | 2855 | 78785 | - | - | 5.56E−02- | 3.87E−03 | - | - | 2858000 | 190400 |
| Model 3 | C | 359943 | 12543 | 46926 | - | - | - | - | 1.40E−02 | 2.13E−02 | 428450 | 30050 |
| Model 4 | D | 439754 | 6455 | 58661 | - | - | 2.39E−02 | 3.54E−03 | 6.64E−04 | 7.83E−03 | 688950 | 247200 |
| Model 5 | E | 392620 | 11581 | 55028 | - | - | 1.03E−02 | 7.73E−03 | - | - | 785100 |  |
| Model 6 | F | 433468 | 6359 | 63517 | 303100 | 59571 | 2.43E−02 | 3.88E−03 | - | - | 2057100 | 390200 |
| Model 7 | G | 378237 | 242659 | 15879 | 9794 | 102283 | - | - | 1.39E−02 | 8.44E−03 | 1087050 | 14700 |
| Model 8 | H | 406633 | 2126 | 22246 | 12471 | 233570 | 7.36E−02 | 7.44E−03 | 3.64E−05 | 7.78E−03 | 649300 | 79900 |

**TABLE S9** Number of candidate SNP loci under putative selection identified by BAYESCENV and RDA.

| **Method** | **SNPs** | **BIO3** | **BIO5** | **BIO11** | **BIO15** |
| --- | --- | --- | --- | --- | --- |
| BAYESCENV | 611 | 174 | 250 | 182 | 289 |
| RDA | 1759 | 627 | 67 | 44 | 1021 |

Note: BIO3 (Isothermality), BIO5 (Max Temperature of the Warmest Month), BIO11 (Mean Temperature of the Coldest Quarter), and BIO15 (Precipitation Seasonality)

**TABLE S10** GO enrichment of environment associated genes (the union data of BAYESCENV and RDA) of *Cupressus gigantea* (*P* < 0.01).

| **GO.ID** | **Term** | **Annotated** | **Significant** | **Expected** | **classicFisher** |
| --- | --- | --- | --- | --- | --- |
| GO:0000413 | protein peptidyl-prolyl isomerization | 77 | 5 | 3 | 6.00E-06 |
| GO:0000398 | mRNA splicing, via spliceosome | 187 | 12 | 8 | 6.70E-06 |
| GO:0000304 | response to singlet oxygen | 13 | 3 | 1 | 9.90E-06 |
| GO:0008150 | Biological process | 2902 | 107 | 118 | 1.60E-05 |
| GO:0001510 | RNA methylation | 18 | 3 | 1 | 2.80E-05 |
| GO:0000381 | regulation of alternative mRNA splicing, via spliceosome | 70 | 4 | 3 | 8.80E-05 |
| GO:0017144 | drug metabolic process | 28 | 3 | 1 | 1.10E-04 |
| GO:0000045 | autophagosome assembly | 30 | 3 | 1 | 1.30E-04 |
| GO:0000302 | response to reactive oxygen species | 107 | 7 | 4.35 | 2.50E-04 |
| GO:0034654 | nucleobase-containing compound biosynthetic process | 55 | 3 | 2.24 | 3.00E-04 |
| GO:1901137 | carbohydrate derivative biosynthetic process | 23 | 4 | 0.93 | 3.70E-04 |
| GO:0006486 | protein glycosylation | 10 | 2 | 0.41 | 4.80E-04 |
| GO:0000911 | cytokinesis by cell plate formation | 49 | 3 | 1.99 | 5.80E-04 |
| GO:0006811 | ion transport | 153 | 5 | 6.22 | 6.40E-04 |
| GO:0000244 | spliceosomal tri-snRNP complex assembly | 12 | 2 | 0.49 | 7.00E-04 |
| GO:0006720 | isoprenoid metabolic process | 14 | 2 | 0.57 | 9.70E-04 |
| GO:0008643 | carbohydrate transport | 16 | 2 | 0.65 | 1.27E-03 |
| GO:0001666 | response to hypoxia | 17 | 2 | 0.69 | 1.43E-03 |
| GO:0009888 | tissue development | 18 | 2 | 0.73 | 1.61E-03 |
| GO:0090407 | organophosphate biosynthetic process | 20 | 2 | 0.81 | 1.99E-03 |
| GO:0006796 | phosphate-containing compound metabolic process | 180 | 7 | 7.32 | 2.02E-03 |
| GO:0006857 | oligopeptide transport | 22 | 2 | 0.89 | 2.41E-03 |
| GO:0000165 | MAPK cascade | 83 | 3 | 3.37 | 2.68E-03 |
| GO:0009693 | ethylene biosynthetic process | 31 | 2 | 1.26 | 4.75E-03 |
| GO:0009416 | response to light stimulus | 35 | 2 | 1.42 | 6.03E-03 |
| GO:0043933 | protein-containing complex subunit organization | 160 | 8 | 6.5 | 6.22E-03 |
| GO:0044281 | small molecule metabolic process | 121 | 3 | 4.92 | 8.10E-03 |
| GO:0000226 | microtubule cytoskeleton organization | 42 | 2 | 1.71 | 8.60E-03 |

**
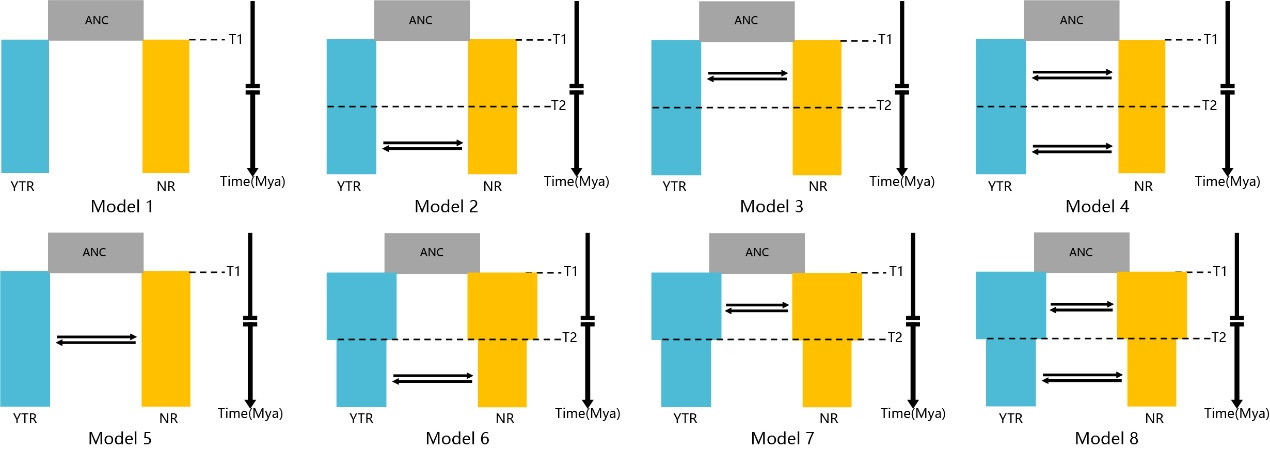
**

**FIGURE S1** Schematic diagram of tested demographic models using FASTSIMCOAL2.


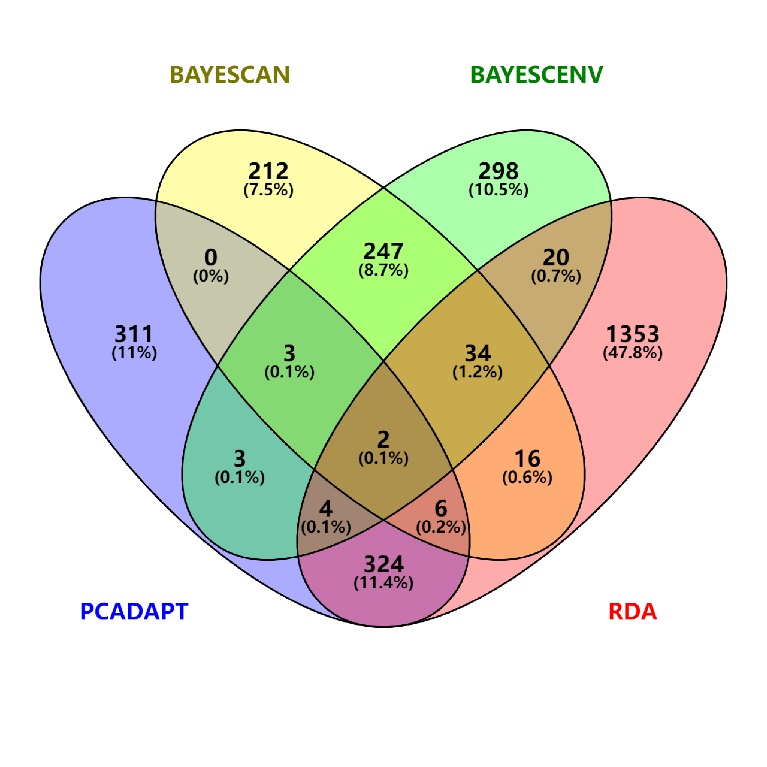


**FIGURE S2** Venn diagram showing the overlap between the total number of SNPs identified as highly differentiated (positive) *F*_ST_-outliers (BAYESCAN and PCADAPT) identified by both methods used (*F*_ST_-outliers) and the number of SNPs significantly associated with at least one climatic variable in two GEA analyses (BAYESCENV and RDA).

**
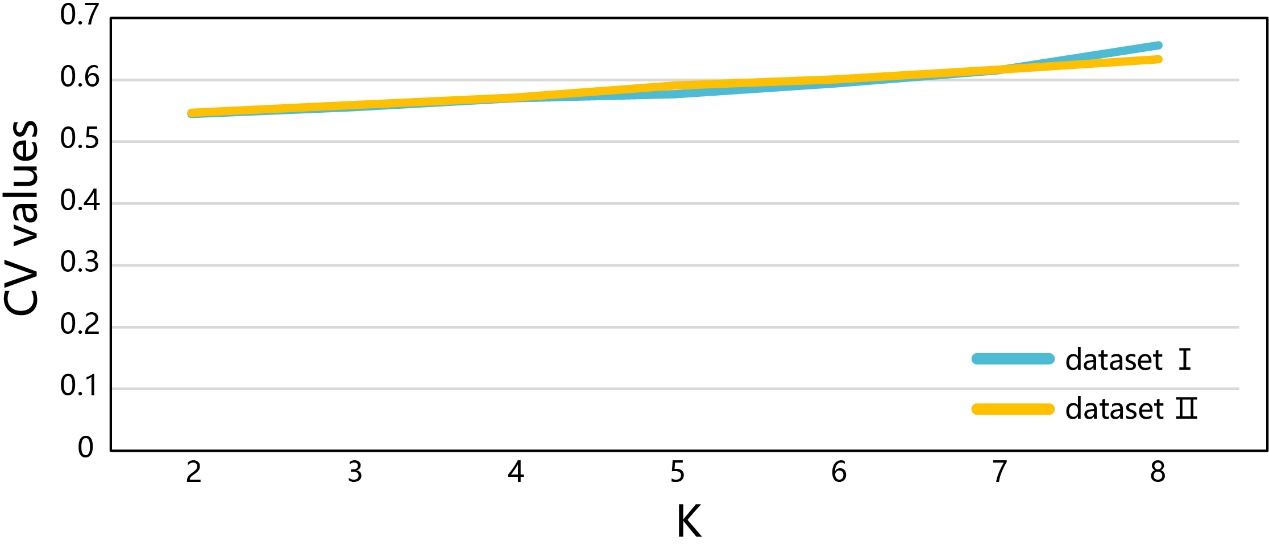
****FIGURE S3** Cross-validation plot for *Cupressus gigantea* in ADMIXTURE analysis based on dataset Ⅰ and Ⅱ.

**
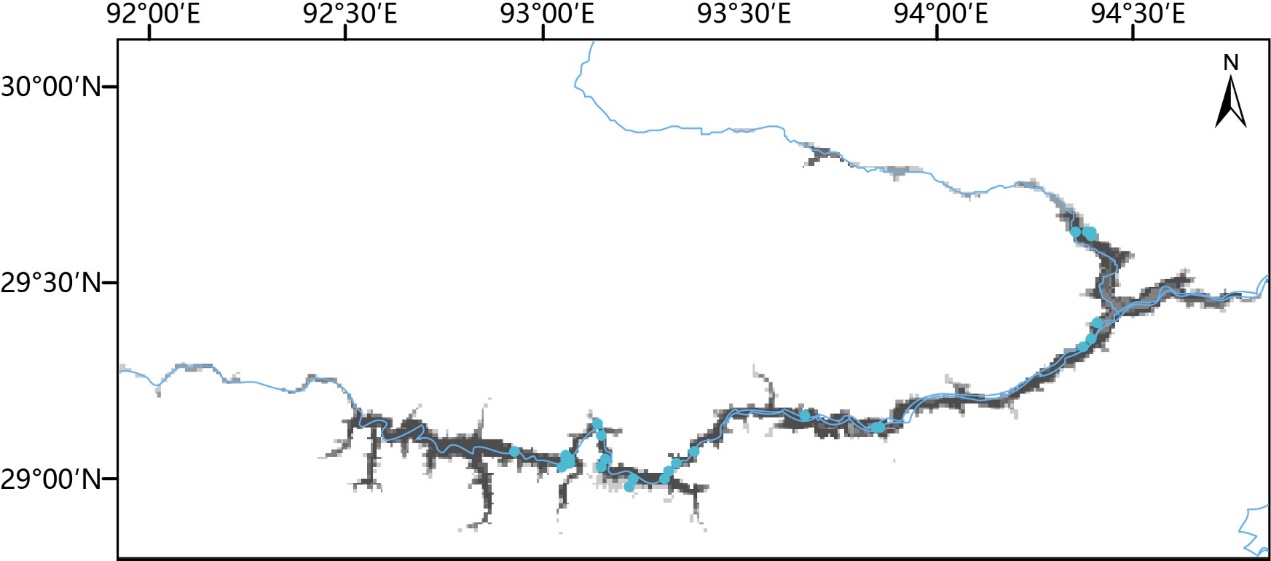
****FIGURE S4** Species distribution models for *Cupressus gigantea*. Potential distributions of *C. gigantea* predicted by BIOMOD2 for current time (1960–1990). The blue circle represents all known occurrence sites according to previous publications and our filed surveys.
